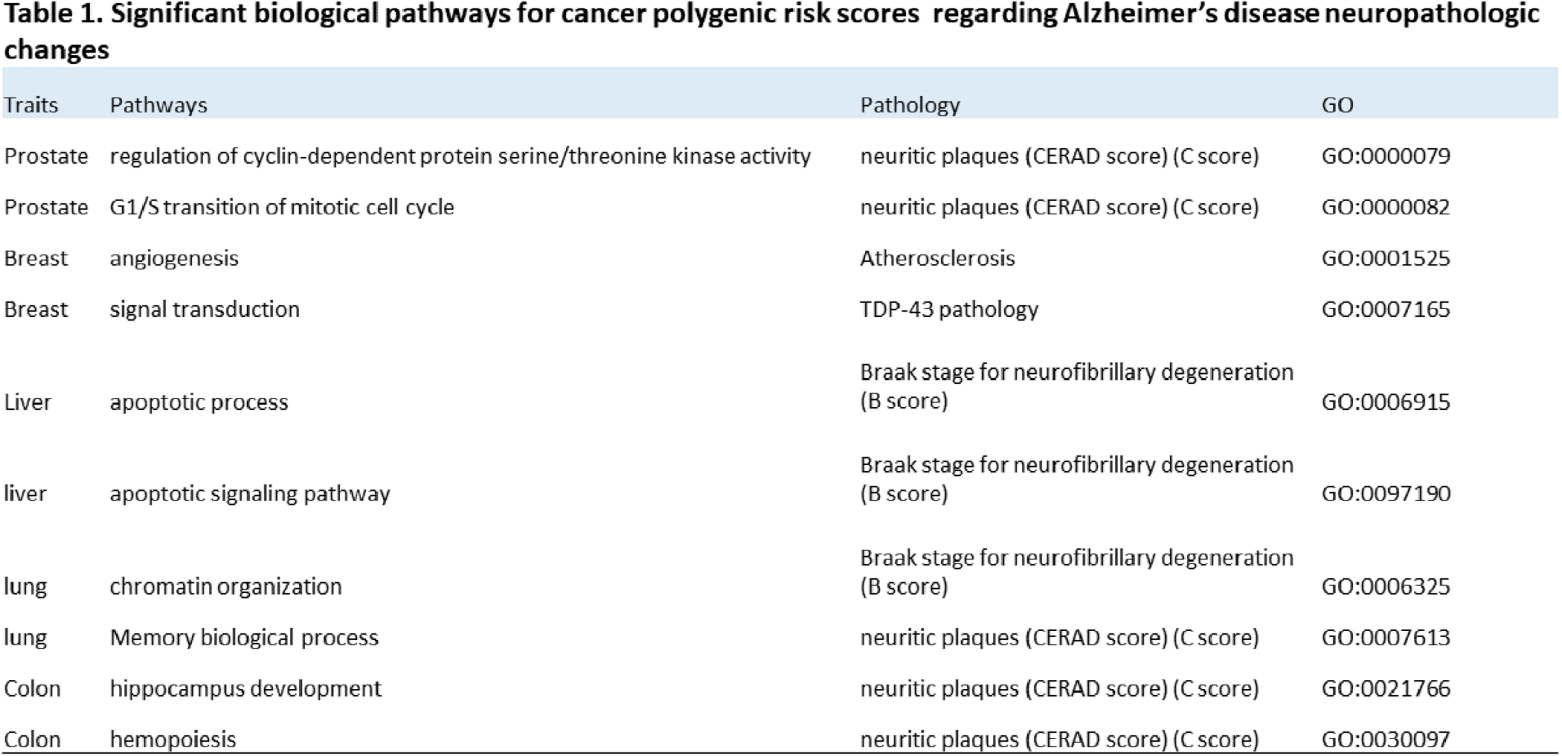# Pathway‐specific polygenic risk score analysis to explore the inverse association between cancer and Alzheimer’s disease neuropathologic changes

**DOI:** 10.1002/alz.089559

**Published:** 2025-01-03

**Authors:** KHINE ZIN AUNG, Xian Wu, Erin L. Abner, Peter T. Nelson, Shama D Karanth, David W. Fardo, Yuriko Katsumata

**Affiliations:** ^1^ Sanders‐Brown Center on Aging, University of Kentucky, Lexington, KY USA; ^2^ College of Public Health, University of Kentucky, Lexington, KY USA; ^3^ University of kentucky, lexington, KY USA; ^4^ University of Kentucky, Lexington, KY USA; ^5^ College of Medicine, University of Florida, Gainesville, FL USA; ^6^ University of Florida, Gainesville, FL USA

## Abstract

**Background:**

Recent research reported that cancer patients had lower risk of Alzheimer’s disease (AD). Common signaling pathways, hormonal systems, and genetic predispositions have been hypothesized as important factors contributing to this inverse association. However, the exact mechanisms are still unknown. Polygenic risk scores (PRS) represent genetic susceptibility and are useful for disease genetic risk prediction. To date, there have been no published findings connecting cancer with AD through PRS. In this study, we investigated the genetic association between various types of cancer and AD/ADRD using biological pathway specific PRS.

**Method:**

We obtained phenotype data from the National Alzheimer’s Coordinating Center (NACC), genotype data from the Alzheimer’s Disease Genetics Consortium (ADGC), and summary statistics for five cancers (liver, lung, prostate, breast, colon cancers) from the United Kingdom (UK) Biobank. We mapped genes to each of the 18,826 Gene Ontology (GO) categories for Homo sapiens and discovered 7,300 biological processes comprised of groups of three or more genes. We removed the APOE region and analyzed the associations between AD and each cancer by using pathway specific PRS. AD neuropathologic changes were evaluated by NACC neuropathology (NP) data including Thal phase, Braak NFT Stage (B0 = no NFTs, B1 = Stage I or ll, B2 = Stage IIl or IV and B3 = V or Vl) and CERAD score (density of neocortical neuritic plaques: C0 = No neuritic plaques, C1 = Sparse neuritic plaques, C2 = Moderate neuritic plaques, C3 = Frequent neuritic plaques).

**Result:**

The most significant biological processes were cell cycle related pathways, apoptotic pathways, angiogenesis, and neurodevelopmental pathways between C scores and B scores (Table 1). Angiogenesis and atherosclerosis pathways were positively significant in TDP‐43 and cerebrovascular pathologies respectively.

**Conclusion:**

While selection and survival bias cannot be completely ruled out, disruptions of specific biological pathways may contribute to the observed inverse relationship of cancer and AD. Further research may clarify the underlying mechanisms between AD and cancer inverse relationship by incorporating with cancer PRS.